# Evolution of Cerebral Ischemia Assessed by Amide Proton Transfer-Weighted MRI

**DOI:** 10.3389/fneur.2017.00067

**Published:** 2017-03-02

**Authors:** Guodong Song, Chunmei Li, Xiaojie Luo, Xuna Zhao, Shuai Zhang, Yi Zhang, Shanshan Jiang, Xianlong Wang, Yuhui Chen, Haibo Chen, Tao Gong, Jinyuan Zhou, Min Chen

**Affiliations:** ^1^Department of Radiology, Beijing Hospital, National Center of Gerontology, Beijing, China; ^2^Graduate School of Peking Union Medical College, Beijing, China; ^3^Department of Radiology, Johns Hopkins University, Baltimore, MD, USA; ^4^Department of Radiology, Zhujiang Hospital of Southern Medical University, Guangzhou, China; ^5^Department of Neurology, Beijing Hospital, National Center of Gerontology, Beijing, China

**Keywords:** APT imaging, chemical exchange saturation transfer imaging, stroke, pH, magnetization transfer

## Abstract

Amide proton transfer-weighted (APTW) magnetic resonance imaging (MRI) has recently become a potentially important tool for evaluating acidosis in ischemic stroke. The purpose of this study was to evaluate the dynamic pH-related changes in the lesions in patients with ischemia. Thirty-nine patients with ischemic stroke (symptom onset to imaging time ranging 2 h–7 days) were examined with a 3.0-T MRI system. Patients were divided into four groups: at the hyperacute stage (onset time ≤ 6 h), at the acute stage (6 h < onset time ≤ 48 h), at the early subacute stage (48 h < onset time ≤ 96 h), and at the late subacute stage (96 h < onset time ≤ 168 h). The APTW signal intensities were quantitatively measured in multiple ischemic regions for each patient. Compared with the contralateral normal white matter, APTW signals were significantly lower in ischemic tissue for all four stages (*P* < 0.05). The APTW signal intensities (APTW_ave_ and APTW_min_) increased consistently with onset time (*R*^2^ = 0.11, *P* = 0.040; *R*^2^ = 0.13, *P* = 0.022, respectively). APTW_max–min_ showed a continued reduction with onset time (*R*^2^ = 0.44, *P* < 0.001). Our results suggest that persistent tissue acidification could occur after ischemia, and as the time from stroke onset increases, the acidotic environment would alleviate. APTW signal intensities could reflect pH-weighted properties in ischemic tissue at different stages and time points.

## Introduction

In the ischemic brain, tissue dysfunction develops from a complex cascade of pathophysiological events that progress temporally and spatially (1, 2). After the critical reduction of cerebral blood flow, normal cerebral oxygen and glucose metabolism are destroyed, and accumulated lactate concentrations due to anaerobic glycolysis often incur intracellular acidosis (3). The maintenance of appropriate intracellular pH in the brain is of paramount importance to its normal physiological activities, for pH regulates various cellular activities and processes (4, 5). As glial acidification is the key trigger of excess glutamate liberation, the glutamate-mediated excitotoxicity can directly induce neuronal cell death (6). Without timely restoration of blood flow, tissue acidification can lead to irreversible tissue damage (7). Intracellular pH of brain tissue varies over ischemic time and cerebral energy status after stroke (8). Thus, tissue pH may serve as a potential surrogate biomarker to reflect the metabolic state and disease evolution during ischemia (9).

Although the use of multimodal magnetic resonance imaging (MRI) protocols in the detection and evaluation of ischemic stroke is increasing (10–12), routine MRI techniques such as perfusion-weighted imaging (PWI) and diffusion-weighted imaging (DWI) are insufficient to depict tissue pH changes, except the magnetic resonance spectroscopy (MRS) method. MRS can detect the changes of cerebral metabolites and measure tissue pH non-invasively, and previous MRS studies have found that early acidosis and subacute alkalosis occurred during ischemic stroke (13, 14). Nevertheless, there still remain challenges for the MRS approach due to low spatial and temporal resolution which limit its clinical application in brain ischemia (15). Thus, novel pH-weighted imaging methods are desired to evaluate the change of intracerebral pH after stroke.

As a variant of the chemical exchange saturation transfer (CEST) MR imaging method (16), amide proton transfer-weighted (APTW) MRI is a novel protein and peptide-based imaging technique (17). By detecting endogenous concentrations of exchangeable amide protons *in vivo*, APTW MRI has been employed to evaluate the changes of mobile abnormal proteins and peptides in tumors (18, 19) and other diseases (20). Besides, it has shown considerable promise in detecting the change in pH non-invasively without any exogenous contrast agents. APTW MRI can assess the severity of tissue acidification and depict the ischemic penumbra by complementing with traditional MRI methods in hyperacute and acute stroke (9, 21–25). To our knowledge, APT-related studies have paid little attention to evaluating dynamic changes of pH in cerebral infarction (26), especially in patients. The clarification of detailed environmental evolutions of the ischemic tissue by APTW MRI would promote our understanding of stroke pathophysiology and may benefit future APT studies in stroke. In this study, changes of tissue pH environment in ischemic stroke are explored using the APTW MRI technique. We will systematically describe APTW MRI signal characteristics in stroke at different phases and investigate their dynamic changes with time, which may facilitate understanding the evolution of ischemic tissue.

## Patients and Methods

### Patients

The study was approved by the local institutional review board. Written informed consent was obtained from all patients participating in this study. Ischemic stroke patients were prospectively and consecutively enrolled during March 2014 to April 2016. The diagnosis of ischemic stroke was based on clinical findings and computed tomography or MRI scans (27). Exclusion criteria included patient’s age <18 years, receiving intravenous t-PA therapy and endovascular thrombectomy before APTW MRI, other brain disorders, unclear symptom onset time, insufficient image quality, small lesions (less than 10 mm in diameter on the transverse DWI), and contraindications to MRI.

### Patient Groups

The time intervals to describe different phases of stroke have not reached general agreement among various researchers (28–30). In our study, 39 patients enrolled were divided into four groups based on the symptom onset to imaging time (28): 4 patients at the hyperacute stage (onset time ≤ 6 h), 18 patients at the acute stage (6 h < onset time ≤ 48 h), 10 patients at the early subacute stage (48 h < onset time ≤ 96 h), and 7 patients at the late subacute stage (96 h < onset time ≤ 168 h).

### MRI Imaging Techniques

Magnetic resonance imaging images were acquired on a 3.0-T MRI system (Achieva, Philips Medical Systems, Best, the Netherlands) with an 8-channel receive-only head coil. Several routine MR images, including axial Diffusion-weighted (DW), T2-weighted, and T1-weighted were collected to confirm the locations of the ischemic stroke lesions and exclude any other structural abnormalities.

Amide proton transfer-weighted MRI was acquired with a fat-suppressed, single-shot, turbo-spin-echo sequence, and the parameters were as follows: repetition time = 3,000 ms, turbo-spin-echo factor factor = 54, acquisition matrix size = 104 × 101, reconstruction matrix size = 400 × 400, field of view = 230 mm × 221 mm, and slice thickness = 6 mm. A multi-offset, multi-acquisition APTW protocol (31, 32) was used, and 31 offsets spanned +6 to −6 ppm [31 offsets = 0, ±0.25, ±0.5, ±0.75, ±1, ±1.5, ±2, ±2.5, ±3.0 (2), ±3.25 (4), ±3.5 (8), ±3.75 (4), ±4 (2), ±4.5, ±5.0, ±6.0 ppm; the values in parentheses were the number of acquisitions, which was 1 if not specified]. An unsaturated image was also acquired for signal normalization. One transverse slice was acquired with the largest ischemic lesions, matching the DWI location. Besides, to evaluate the conventional magnetization transfer effect, a saturated image at 15.6 ppm was also acquired. The total duration of the APTW MRI sequence was 3 min 12 s.

### Data Processing and Analysis

We used the Interactive Data Language (Exelis Visual Information Solutions, Boulder, CO, USA) to process the APTW MRI data. The pH-sensitive CEST effect was detected by analyzing the z-spectrum on a pixel-by-pixel basis (17). In the z-spectrum, the amide proton frequency was conventionally referenced with respect to the water signal (assigned to be 0 ppm). Magnetization transfer ratio was defined as MTR = 1 − *S*_sat_/*S*_0_ (in which *S*_sat_ and *S*_0_ were the signal intensities with and without selective RF irradiation), and MTR asymmetry (MTR_asym_) analysis was performed with respect to the water signal. Thus, the APTW signal was calculated based on the MTR_asym_ at 3.5 ppm, i.e., MTR_asym_(3.5 ppm) = *S*_sat_(−3.5 ppm)/*S*_0_ − *S*_sat_(+3.5 ppm)/*S*_0_. Of course, MTR_asym_(3.5 ppm) contains not only the mobile amide proton transfer ratio (APTR), which is related to pH and other changes in tissue, but also the complicating ${\text{MTR}}_{\text{asym}}^{’}(3.5\text{ ppm})$, resulting in ${\text{MTR}}_{\text{asym}}(3.5\text{ppm})=\text{APTR}+{\text{MTR}}_{\text{asym}}^{’}(3.5\text{ ppm})$. ${\text{MTR}}_{\text{asym}}^{’}(3.5\text{ ppm})$ can be attributed to the upfield nuclear Overhauser enhancement (NOE) effect of various non-exchangeable protons (33, 34). Thus, MTR_asym_(3.5 ppm) images are generally called APTW images.

**Figure 1:**
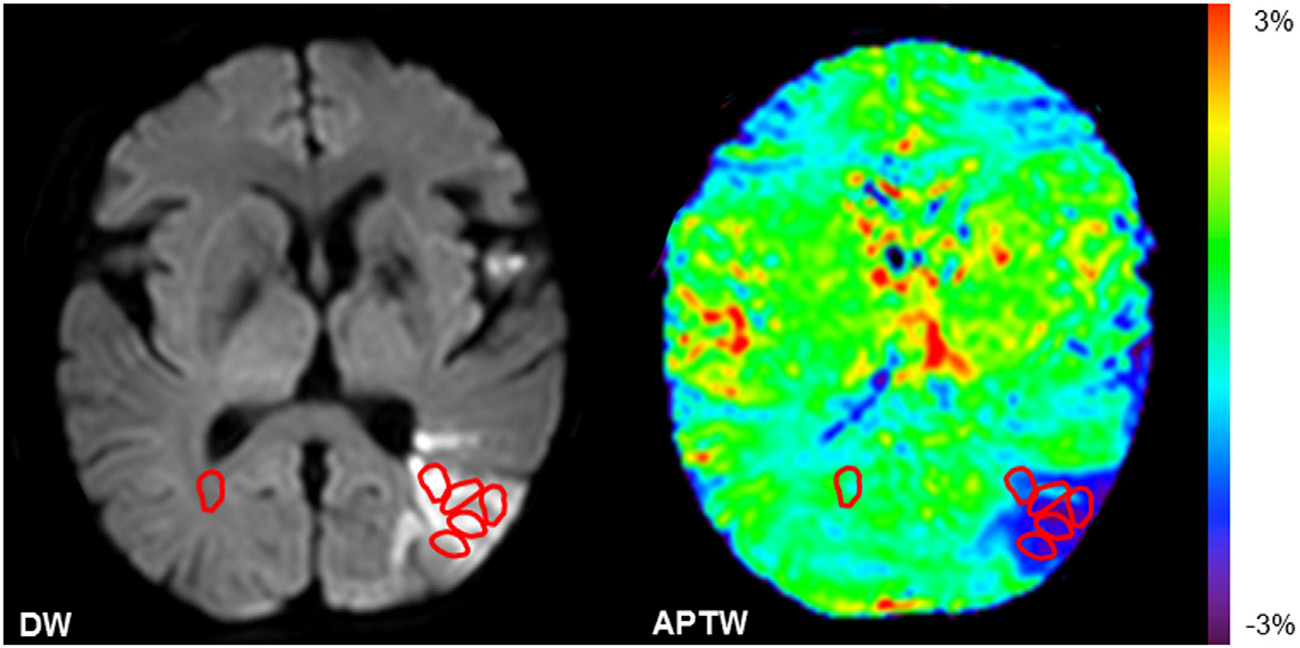
**Examples of the definition of the regions of interest (ROIs)**. Five ROIs in the ischemic tissue and one ROI in the contralateral normal white matter were chosen.

The quantitative MRI analysis was performed in five small regions of interest (ROIs) within the ischemic lesions defined by DWI for each patient (Figure 1). One ROI in the contralateral normal white matter (CNWM) was also chosen for comparison. The maximum APTW (APTW_max_) signal intensity, the minimum APTW (APTW_min_) signal intensity, and the difference between APTW_max_ and APTW_min_ (APTW_max–min_), which reflects APTW signal heterogeneity, the average APTW (APTW_ave_), and MTR(15.6 ppm) signal intensity (corresponding to the APTW_min_), were reported. In the contralateral normal tissue, APTW (APTW@CNWM) and MTR(15.6 ppm) signal intensities were also calculated.

### Statistical Analysis

All statistical analyses were performed using the SPSS 17.0 software package. APTW and MTR signal intensities in the ischemic tissue and the contralateral normal tissue were obtained for each patient. The results were presented as the format of mean ± SE. The difference of sex among the four groups was compared using Chi-squared test. Time-related changes of APTW signals were assessed by regression analysis, and goodness-of-fit was determined by calculation of an R^2^ value. One-way analysis of variance with *post hoc* tests was performed for comparing multiple values of parameters at different stages. Tukey’s *post hoc* tests were used if the *P* value resulted from tests for homogeneity of variance was greater than or equal to 0.05. Otherwise, Games-Howell *post hoc* tests would be employed if *P* < 0.05. The comparisons of MTR parameters between the ischemic tissue and CNWM were analyzed by Student’s *t*-test. *P* values <0.05 were considered statistically significant.

## Results

### Baseline Data

A total of 62 patients were recruited and 39 patients remained in our study. Twenty-three patients were ineligible because of small ischemic lesions on the routine DW images (*n* = 10), accompaniment with brain tumors (*n* = 2), an unclear onset time (*n* = 4), obvious motion artifacts (*n* = 5), and the intravenous t-PA treatment before APTW MRI (*n* = 2). Of the 39 included patients, baseline data are shown in Table 1. These data show no significant sex or age-related differences, while there were obviously significant differences of the mean onset to scan time among four stages.

**Table 1:** **Baseline demographic data for patients with different stages**.

Variable	Hyperacute stage (0–6 h)	Acute stage (6–48 h)	Early subacute stage (48–96 h)	Late subacute stage (96–168 h)	*P* value
Age (years)^a^	63 ± 6	66 ± 4	58 ± 4	61 ± 2	0.587
Male/female	2/2	10/8	6/4	5/2	0.769
Onset time (h)^a^	4.5 ± 1.0	31.5 ± 2.9	79.2 ± 3.7	147.4 ± 6.3	<0.001

*^a^Mean value ± SE*.

### MTR_asym_ Spectrum and APTW Image Features

**Figure 2:**
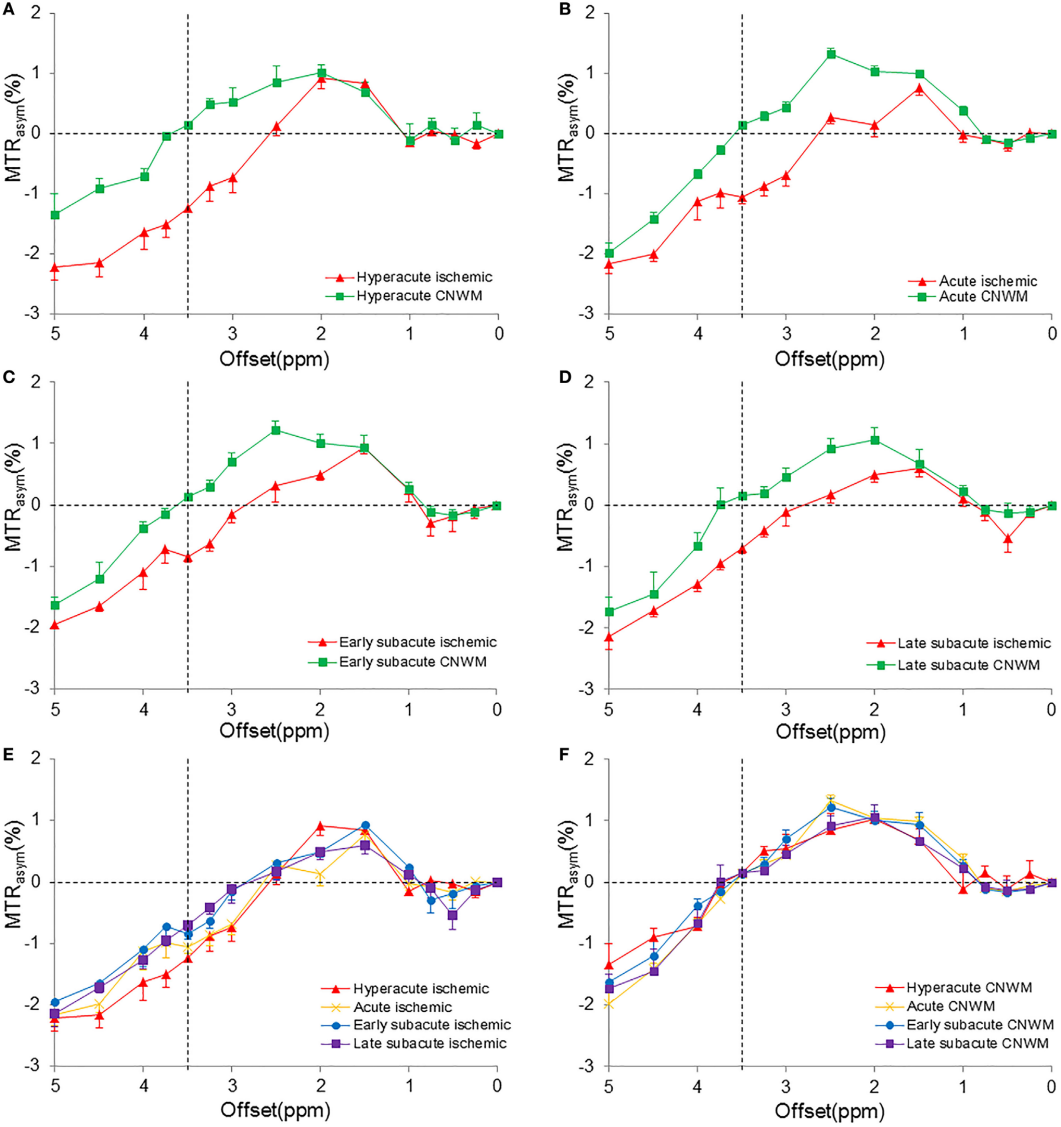
**Measured MTR_asym_ spectra in the ischemic tissue and the contralateral normal white matter (CNWM) of different stages (corresponding to the APTW_min_ value)**. **(A)** Hyperacute stage. **(B)** Acute stage. **(C)** Early subacute stage. **(D)** Late subacute stage. **(E)** The combination of MTR_asym_ spectra in the ischemic tissue of different stages. **(F)** The combination of MTR_asym_ spectra in the CNWM of different stages. The chemical exchange saturation transfer effect reduced in the offset range of 2–5 ppm in all stages. MTR_asym_(3.5 ppm) showed the largest reduction in the hyperacute stage **(A)**. An increase was shown in the MTR_asym_(3.5 ppm) in the ischemic tissue **(E)**.

Figure 2 shows the average MTR_asym_ spectra corresponding to APTW_min_ values of the four stages. For all patients enrolled, the CEST effect in the ischemic tissue showed a visible reduction at the offset range of 2–5 ppm in the MTR_asym_ spectra compared with the CNWM. All MTR_asym_ spectra became negative at offsets greater than 3 ppm (in the ischemic areas) or >3.5 ppm (in the CNWM), as reported previously (35, 36). The maximal CEST effect reduction appeared at the offset of 3.5 ppm, where amide resonances of the backbones of soluble proteins and peptides were present, and the process has been proved to be sensitive to the change of pH (17). Among different stages, MTR_asym_(3.5 ppm) signal intensities, which were the apparent APTW signals, showed larger reduction during the hyperacute stage than the other stages. A trend toward higher values of MTR_asym_(3.5 ppm) signal intensities in the ischemic tissue could be observed among the four stages after stroke (Figure 2E). On the contrary, MTR_asym_(3.5 ppm) values in the CNWM stayed stable (Figure 2F).

**Figure 3:**
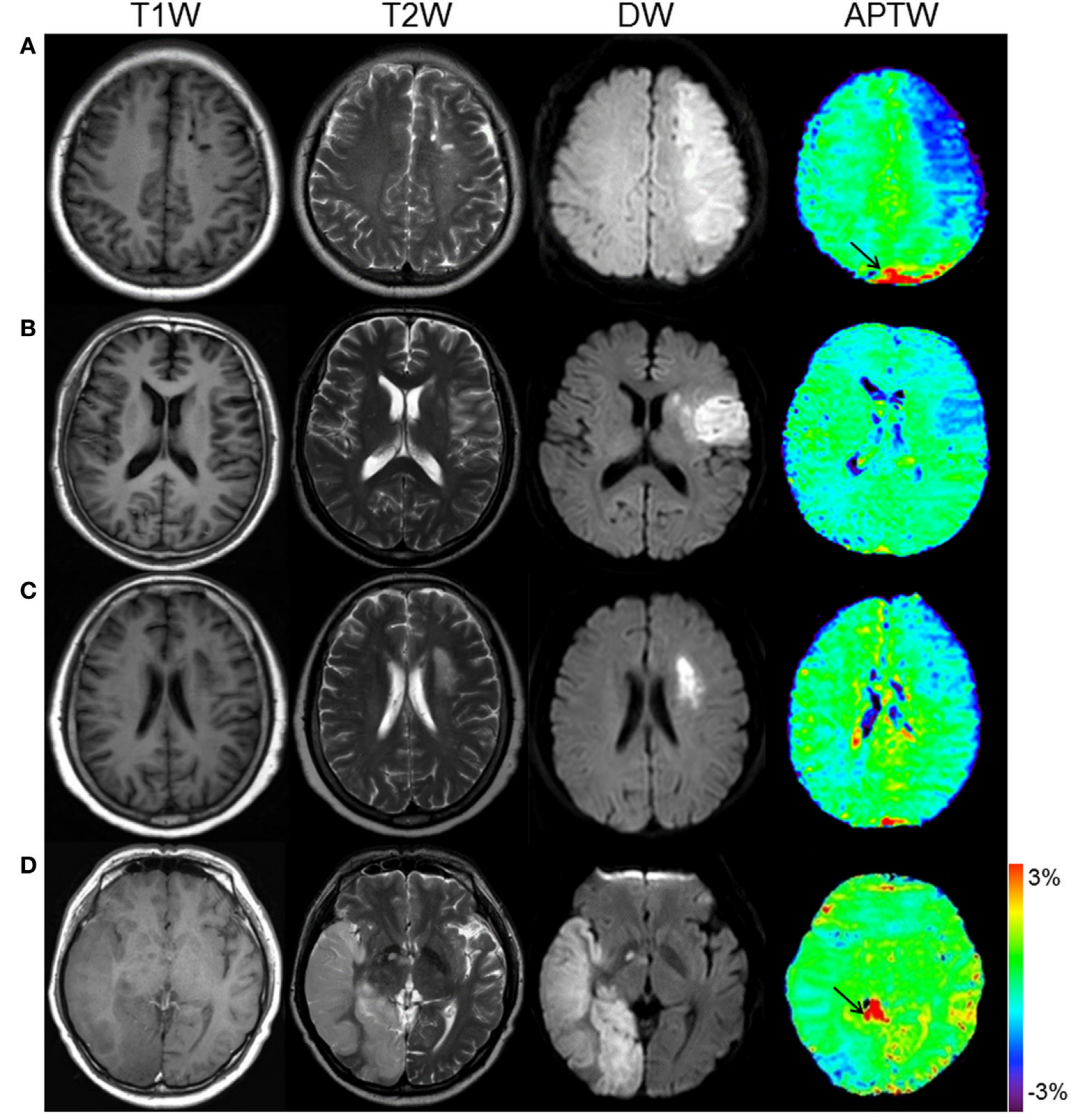
**APT and conventional MR images for different stage patients**. **(A)** F/50 years, a hyperacute stage patient at 2 h after symptom onset. APTW_ave_ = −0.83%, APTW_max–min_ = 1.30%. **(B)** M/54 years, an acute stage patient at 17 h after symptom onset. APTW_ave_ = −0.63%, APTW_max–min_ = 0.99%. **(C)** F/62 years, an early subacute stage patient at 72 h after symptom onset. APTW_ave_ = −0.40%, APTW_max–min_ = 0.72%. **(D)** M/54 years, a late subacute stage patient at 120 h after symptom onset. APTW_ave_ = 0.03%, APTW_max–min_ = 0.53%. Note the presence of CSF artifacts (black thin arrows).

Figure 3 shows the APTW and standard MR images of patients at four different stages. From the images, we can see that APTW signal intensities in the ischemic tissue were visibly lower than the CNWM at the hyperacute stage, the acute stage, and the early subacute stage. However, APTW images acquired from patients at the late subacute stage showed that the reduction of APTW values in the ischemic tissue was not obvious compared with those in CNWM (APTW@CNWM), which suggested pH restoration might occur.

### Quantitative Analyses of APTW and MTR Signal Intensities

Tables 2 and 3 quantitatively compare several APTW and MTR parameters at different stages post-stroke. APTW_ave_, APTW_min_, and APTW_max_ values were significantly lower in the ischemic tissue than APTW@CNWM (*P* < 0.05 in all four stages), which suggested a reduction of APT effect compared with the CNWM occurred after ischemia. APTW_ave_ and APTW_max_ values showed no significant differences among different stages, which suggested that persistent acidification might exist in the four different stages. In addition, APTW_min_ values in the hyperacute stage were significantly lower than the early and late subacute stage (*P* = 0.006, and *P* = 0.003, respectively), suggesting tissue acidification in the ischemic tissue might be more severe in the hyperacute stage.

**Table 2:** **Amide proton transfer-weighted (APTW) intensity values among four stages (%; mean value ± SE)**.

Variable	Hyperacute stage	Acute stage	Early subacute stage	Late subacute stage
APTW_ave_	−0.85 ± 0.02	−0.64 ± 0.09	−0.48 ± 0.11	−0.34 ± 0.10
APTW_max_	−0.02 ± 0.04	−0.24 ± 0.07	−0.23 ± 0.10	−0.11 ± 0.07
APTW_min_	−1.24 ± 0.04	−1.06 ± 0.10	−0.85 ± 0.08	−0.70 ± 0.09
APTW_max–min_	1.22 ± 0.06	0.82 ± 0.07	0.62 ± 0.05	0.59 ± 0.07
APTW@CNWM	0.15 ± 0.01	0.14 ± 0.01	0.14 ± 0.02	0.16 ± 0.01
*t*-Test *P* values^a^	**<0.001, 0.014, <0.001**	**<0.001, <0.001, <0.001**	**0.001, 0.019, <0.001**	**0.008, 0.030, <0.001**

*^a^Three *post hoc P* values corresponded to those between APTW_ave_ (or APTW_max_ or APTW_min_) and APTW@CNWM. Bold indicates a significant change*.

**Table 3:** **MTR(15.6 ppm) intensity values in the ischemic lesion and the contralateral normal white matter (CNWM) among four stages (%; mean value ± SE)**.

Variable	Hyperacute stage	Acute stage	Early subacute stage	Late subacute stage	Analysis of variance *P* value
Lesion	26.61 ± 0.89	26.30 ± 0.99	25.39 ± 1.08	23.63 ± 2.58	0.625
CNWM	31.14 ± 1.15	31.08 ± 0.77	30.91 ± 1.92	29.36 ± 1.89	0.840
*t*-Test *P* values^a^	**0.031**	**<0.001**	**<0.001**	**0.036**	

*^a^*t*-Test *P* values corresponded to the results between MTR(15.6 ppm) values in the ischemic lesion and the CNWM. Bold indicates a significant change*.

APTW_max–min_ was significantly higher in patients at the hyperacute stage after stroke than in patients at the other three stage (*P* = 0.006, *P* = 0.001, and *P* = 0.001, respectively), suggesting that the most heterogeneous APTW signal variety existed at the hyperacute stage of stroke. In contrast, APTW@CNWM values among groups showed no significant difference.

Paired Student’s *t*-test results showed that the MTR(15.6 ppm) value in the ischemic tissue was significantly lower compared with that in CNWM at the four stages (*P* = 0.031, *P* < 0.001, *P* < 0.001, and *P* = 0.036, respectively), indicating that the conventional MT effect may be reduced after ischemia. In addition, multiple comparisons of MTR(15.6 ppm) values in the ischemic tissue among groups showed no significant differences, and similar results were found for MTR(15.6 ppm) values in the CNWM.

### Regression Analysis of APTW Signal Intensities with Time

**Figure 4:**
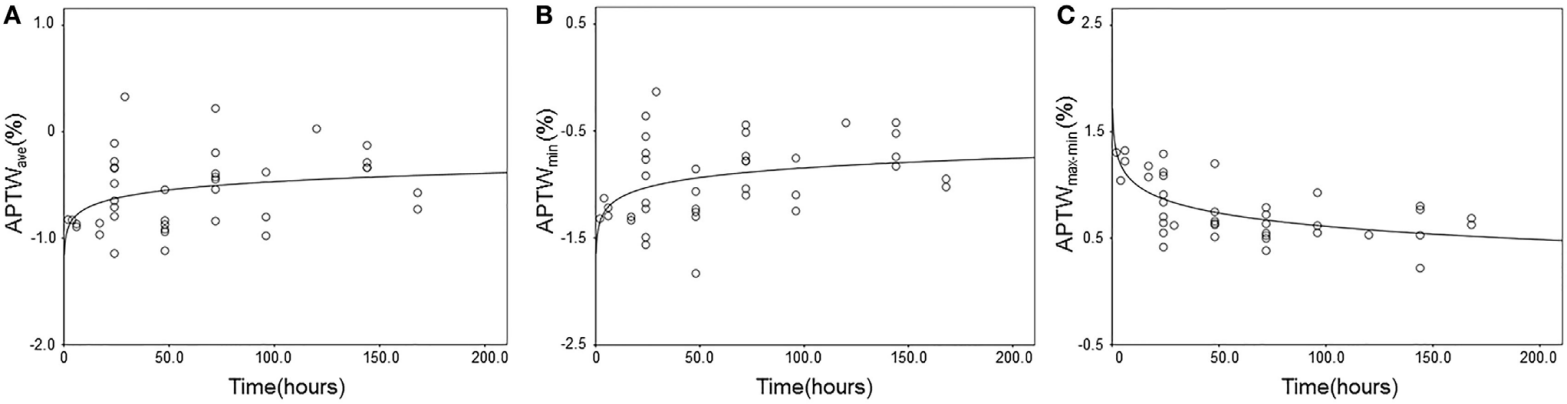
**Experimental and fitted time-related changes of different amide proton transfer-weighted (APTW) values in the ischemic tissue**. **(A)** APTW_ave_ values (*R*^2^ = 0.11, *P* = 0.040); **(B)** APTW_min_ values (*R*^2^ = 0.13, *P* = 0.022); **(C)** APTW_max–min_ values (*R*^2^ = 0.44, *P* < 0.001).

Figure 4 shows the results of regression analysis of APTW signals against the onset time. There were significantly increasing logarithmic time-related changes of the APTW_ave_ and APTW_min_ values (*R*^2^ = 0.11, *P* = 0.040; *R*^2^ = 0.13, *P* = 0.022, respectively), which indicated that tissue acidification alleviated with time, but the goodness-of-fit was relatively poor. In addition, APTW_max–min_ values followed a decreasing logarithmic curve with the onset time (*R*^2^ = 0.44, *P* < 0.001), indicating that the APTW signal heterogeneity in the ischemic tissue reduced with time, and the most significant changes seemingly occurred within the first few hours. In contrast, no significant time-related change was observed for APTW_max_ and APTW@CNWM values.

## Discussion

In this study, we used the APTW MRI technique to investigate the dynamic change of pH in ischemic tissue in patients at the early stage after stroke (the onset to scan time varied from 2 h to 7 days), which has not been the focus clinically before. We found that tissue acidification in the ischemic zone would occur after stroke compared with the contralateral normal tissue, and the acidification would alleviate with the onset time increased. The APTW intensity showed higher heterogeneity at the hyperacute and acute stage after stroke, which suggested that pH variety existed during this time period. This was consistent with the finding of graded ischemic acidosis in previous studies (24, 25). The APTW intensity became more homogeneous as the onset to scan time increased. As APTW signals might be affected by potential confounding influences of treatment effects, we acquired APTW images in patients without receiving interventions of intravenous t-PA therapy and endovascular treatment, which would help us to describe the dynamic change of APT effect in the progress of stroke without treatment interventions.

We studied several APT parameters (APTW_ave_, APTW_max_, APTW_min_, APTW_max–min_, and the CNWM APTW signal intensities) in patients at different stages after stroke and evaluated time-related changes of the APTW effect by regression analysis. The results showed that APTW_ave_ and APTW_min_ signal intensities acquired in the infarction lesions were reduced significantly in the ischemic tissue compared with those in the CNWM at the hyperacute and acute stages, suggesting that tissue acidosis occurred after stroke onset, which had been confirmed by previous studies during the initial ischemic period of stroke (17, 37). The reduction of intracellular pH is mainly due to the accumulation of lactic acid in anaerobic glycolysis, and the correlation of APT effect with intracellular pH has been proved (22, 26). In addition, APTW_ave_ and APTW_min_ values were also lower compared with those in the CNWM at the subacute stage, which may suggest that APTW effect could still be reduced during this time period, and the results are consistent with Zhao’s study (38). In their study, they just found hypointense APTW signals in a small number of stroke patients. As tissue acidification is one of the important pathophysiology factors affecting APTW signals (26), the reduction of APTW_ave_ and APTW_min_ signals during this stage may be partially due to tissue acidification. The results suggested that persistent tissue acidification might occur after stroke.

Moreover, we found that APTW_ave_ and APTW_min_ values were lower at the hyperacute stage, and higher at the subacute stage than the other stages, and the regression analysis demonstrated that APTW_ave_ and APTW_min_ signals intensities increased with the onset to scan time. The increase of APTW_ave_ and APTW_min_ suggested that acidosis of the infarction tissue may be reduced and alleviated with the onset to scan time, and this is consistent with several previous studies (13, 14), which have showed that the reduced pH of ischemic brain would increase after stroke in the follow-up of several patients by the MRS method. Similar results have been detected in animal models by the APTW MRI method (26). The increase of pH after stroke could be explained by active compensatory mechanisms within the ischemic tissue according to prior results (14). In addition, a shift from acute acidosis to subacute alkalosis was detected in several works (13, 14, 39, 40), and alkalotic pH occurred in the first few days after ischemia could be influenced by the duration and degree of ischemia acidosis (41). We did not consistently observe the shift in our current data; the deviation of the pH changes might be contributed to the confounding factors that may influence the APT imaging results in ischemic tissue, such as the tissue temperature (26) and protein concentration (17).

Previous studies have shown that the infarct core suffered severe acidosis, and the peri-infarct tissue suffered from moderate acidification (25, 42). The heterogeneity of the APTW signal (APTW_max–min_) may reveal the diversity of pH values in the ischemic tissue. From the fitted curve, it showed that APTW_max–min_ decreased significantly with the onset to scan time of stroke symptoms. The dynamic change of APTW_max–min_ suggests that the ischemic tissue seems to become more homogeneous with the increase of onset to scan time. The seemingly significant changes occurred during the first few hours after stroke, which corresponded to the hyperacute stage, and patients at this stage had significantly higher APTW_max–min_ values than other stages. This period is consistent with the therapeutic time window for stroke. APTW_max–min_ may serve as a useful biomarker to reflect tissue microenvironment at different time points after ischemia and allow further options of stroke interventions.

In addition, APTW signals of the CNWM were stable in the four stages, which suggested relatively unremarkable interindividual variability of the APTW@CNWM values among different individuals. The magnetization transfer effect in the ischemic tissue reduced comparing with the CNWM, which may contribute to the cerebral edema and partial neuronal death after ischemia. Consistent with previous studies, our results for the magnetization transfer effect remained stable in the first several days after stroke (43, 44). The inconsistency between APTW and MTR values in the ischemic tissue suggests APT effect may change gradually without the influence of underlying MT effects at the early stage after stroke.

Our research shows some preliminary results of pH-weighted APT effect in ischemia, which may be helpful for further APTW MRI studies in stroke. Here, we mainly focus on patients without the consideration of therapeutic interventions, and further studies are needed to clarify the detailed APT effect changes after receiving treatment. Furthermore, due to a very limited number of patients receiving PWI in our study, we did not take PWI into consideration, which limited the full evaluation of penumbra. The number of subjects in our study was relatively small especially at the hyperacute phase, and patients just received one scan without longitudinal follow-up APTW MRI data. Thus, a large-scale longitudinal study including more patients and scan time points would be required to validate our results. In addition, we used single slice image acquisition, and APTW signals in other ischemic lesions could not be evaluated. Therefore, the three-dimensional APT imaging method, which has been developed in gliomas (45), should be optimized and validated in future stroke studies.

Finally, the standard APTW metric, MTR_asym_(3.5 ppm), was used in this study. As reported previously (35, 36), all MTR_asym_ spectra were negative at the higher offsets (>3 ppm for the ischemic areas or >3.5 ppm for the CNWM) due to the possible inherent asymmetry of the conventional MT effect and the possible NOEs of aliphatic protons of mobile macromolecules (33, 34). It has been demonstrated recently that MTR_asym_(3.5 ppm) remains a valid metric for APT imaging at 3 T (46, 47). However, it is essentially important to obtain relatively pure APT and NOE signals and to assess whether both are pH dependent (48–50). In this regard, a novel APT imaging analysis approach (46, 47), such as the extrapolated semisolid magnetization transfer reference, may be used.

In conclusion, our study shows that tissue acidification after stroke may alleviate as the onset to scan time increases. APTW signal intensities could reflect pH-weighted properties in ischemic tissue at different stages and time points. APTW MRI provides an alternative method to depict pH changes in ischemia, and APTW signals could potentially serve as a surrogate pH-weighted imaging marker in non-invasive and dynamic evaluation of tissue evolution in patients at the early stage after stroke.

## Ethics Statement

This study was carried out in accordance with the recommendations of the human ethics committee of the Beijing Hospital with written informed consent from all subjects. All subjects gave written informed consent in accordance with the Declaration of Helsinki. The protocol was approved by the human ethics committee of the Beijing Hospital.

## Author Contributions

JZ and MC conceived and designed the research. GS, CL, XL, XZ, SZ, YZ, SJ, XW, YC, HC, and TG performed the research. All the authors analyzed the data. GS, JZ, and MC wrote the paper. CL, JZ, and MC obtained funding. All the authors read and approved the final draft.

## Conflict of Interest Statement

The authors declare that the research was conducted in the absence of any commercial or financial relationships that could be construed as a potential conflict of interest. The reviewer PR and handling editor declared their shared affiliation and the handling editor states that the process nevertheless met the standards of a fair and objective review.
